# The Immune Epitope Database (IEDB): 2018 update

**DOI:** 10.1093/nar/gky1006

**Published:** 2018-10-24

**Authors:** Randi Vita, Swapnil Mahajan, James A Overton, Sandeep Kumar Dhanda, Sheridan Martini, Jason R Cantrell, Daniel K Wheeler, Alessandro Sette, Bjoern Peters

**Affiliations:** 1 La Jolla Institute for Allergy and Immunology, Division of Vaccine Discovery, La Jolla, CA 92037, USA; 2 Knocean Inc., Toronto, Ontario M2P 2T3, Canada; 3 Leidos Health, LLC, San Diego, CA 92121, USA; 4 University of California San Diego, Department of Medicine, La Jolla, CA 92093, USA

## Abstract

The Immune Epitope Database (IEDB, iedb.org) captures experimental data confined in figures, text and tables of the scientific literature, making it freely available and easily searchable to the public. The scope of the IEDB extends across immune epitope data related to all species studied and includes antibody, T cell, and MHC binding contexts associated with infectious, allergic, autoimmune, and transplant related diseases. Having been publicly accessible for >10 years, the recent focus of the IEDB has been improved query and reporting functionality to meet the needs of our users to access and summarize data that continues to grow in quantity and complexity. Here we present an update on our current efforts and future goals.

## INTRODUCTION

Established in 2004, the Immune Epitope Database (IEDB) contains >1.6 million experiments representing the adaptive immune response to epitopes, gathered primarily from the literature (1). These experiments were manually curated following structured curation guidelines, as previously described (2). This data was obtained from 19 500 publications and includes all the literature available from the beginnings of PubMed until now. Historical curation of papers going back to 1952 was completed in 2011 and since, we have focused on newly published papers. We perform a query of PubMed every two weeks to remain current with new content. The IEDB has approximately 300 unique visitors and 1220 page views per day. The IEDB exists as a free service with the goal of helping further immunological research. Thus, we routinely perform outreach activities to interact with our users to ascertain their needs and gather feedback on existing features. Here we present our efforts toward meeting user needs, as well as extending functionality to keep current with accepted web standards.

**Figure 1.**
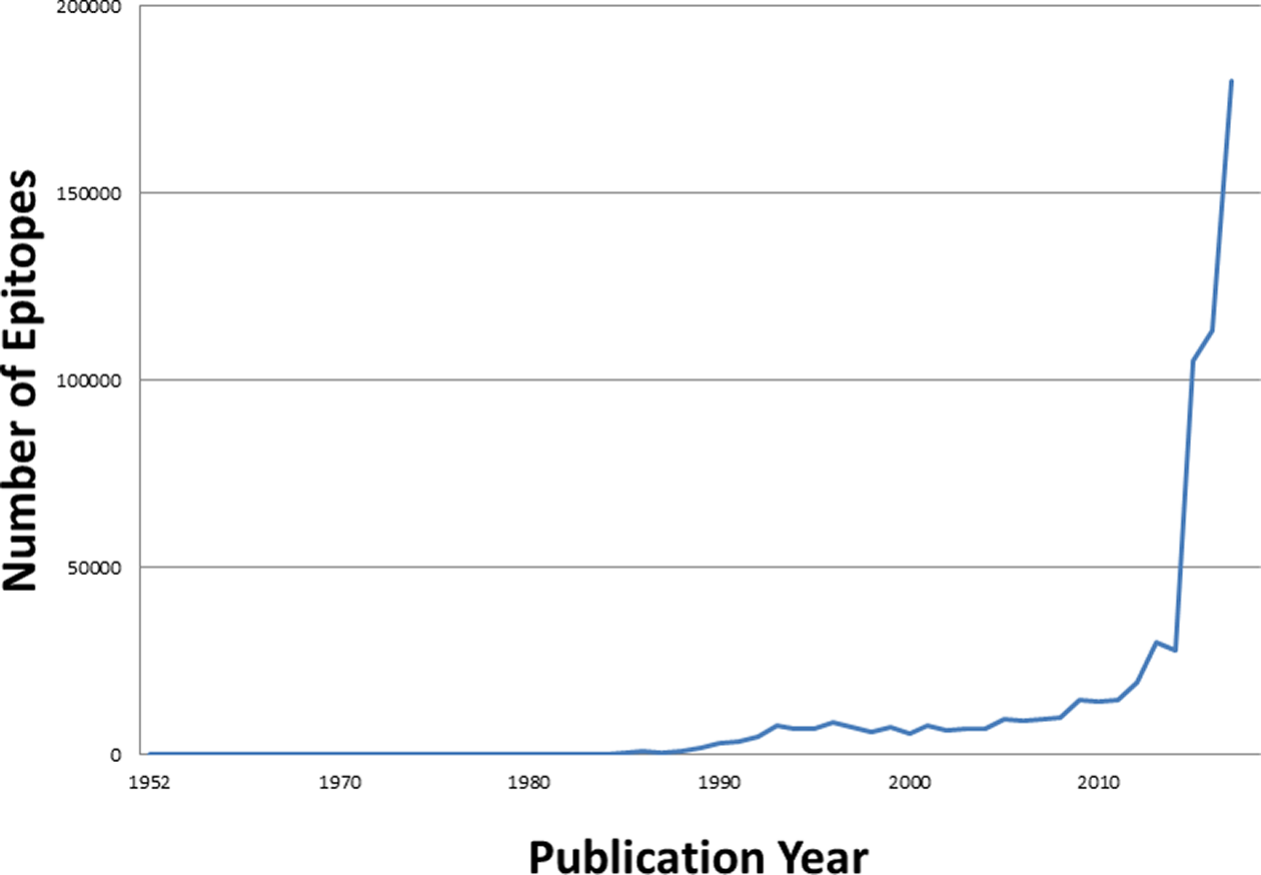
Number of epitopes curated by year. A rapid increase in the number of epitopes curated for each year of publication is due to authors increasingly publishing very large datasets.

Significantly, research is ever-evolving; new experiments are continually created, expanding data quantity and complexity. As the cost of high throughput experiments is decreasing, scientists are publishing greater numbers of experiments per publication, leading to rapid increases in our data. This is reflected in the number of epitopes curated per publication year, which began rapidly increasing in 2015, as shown in Figure 1. Accordingly, the number of experiments captured in the IEDB has also increased by 140% since 2015, now surpassing 1.6 million.

Another factor leading to large amounts of new data is the addition of receptor sequence data to the IEDB schema. Previously, we only captured full length antibody and T cell receptor (TCR) sequences whenever a 3D structure was available, but we now capture both full length and CDR sequences, as well as gene usage whenever authors provide this. To accommodate this new data, we added new database tables, search panes, results tabs, and details pages, as described in a separate publication (Mahajan, *et al*, submitted).

## OUTREACH

To best serve the scientific community, we rely heavily on feedback from our users. We collect user questions and concerns via an online helpdesk feature, a hosted IEDB booth at four national conferences per year, and our annual user workshop, consisting of two days of intensive interaction with a diverse group of users, including students, established investigators, and industry professionals. Lastly, we annually perform an analysis of website usage statistics and query logs to evaluate actual user behavior. Each year, the totality of this feedback is compiled to prioritize improvements to the IEDB, with a focus on the search interface and presentation of search results.

## SEARCH INTERFACE

**Figure 2.**
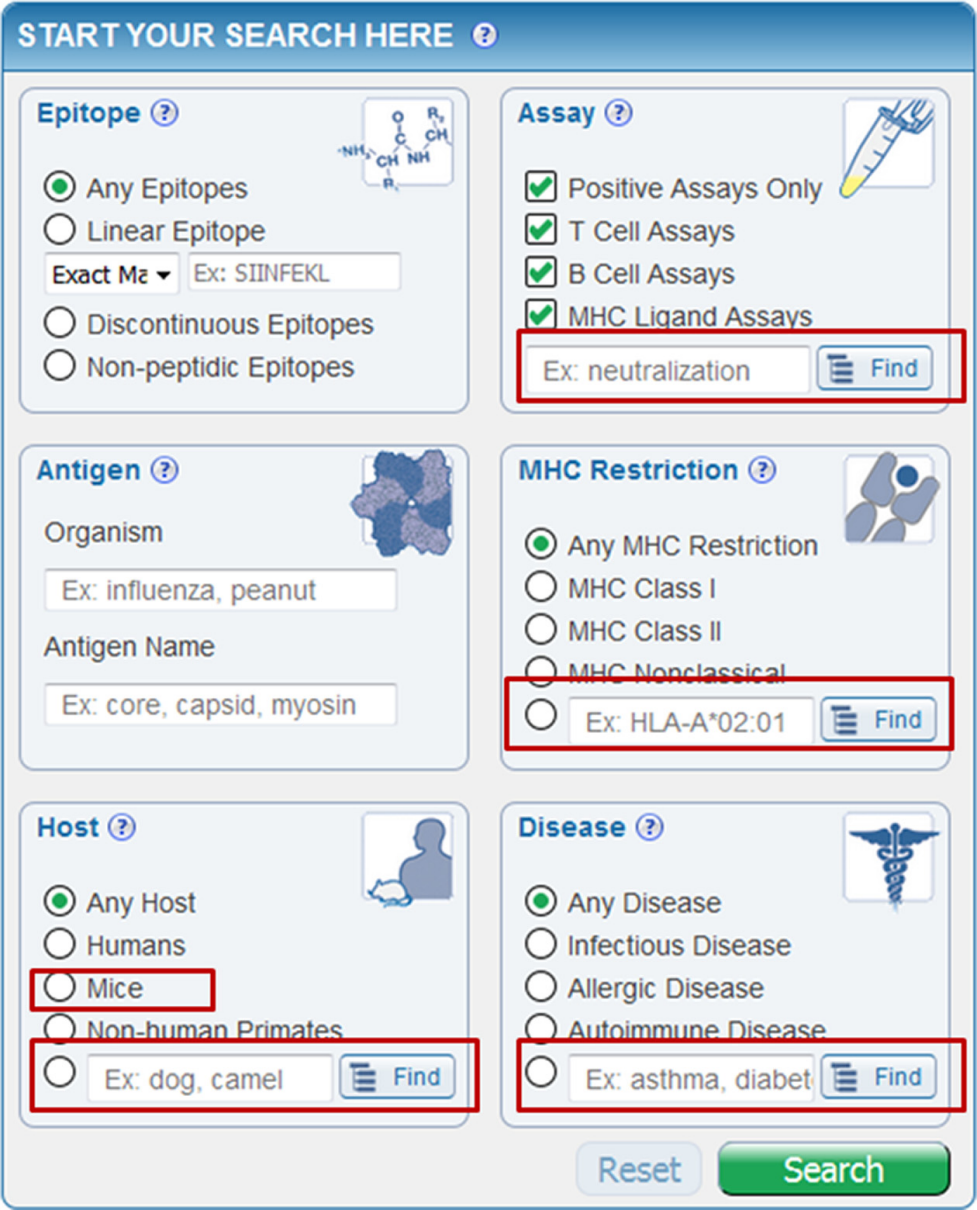
Redesigned home page search interface. New search features (highlighted by red boxes) were designed based on user feedback and analysis of search behaviors.

In 2014, we performed a major redesign of the search interface (1). To examine how well it met the needs of users, and how it could be further optimized, we analyzed query logs from 2016. We found that most queries utilized a single field, and most users searched for a specific linear epitope sequence. This was a positive finding, as this field is the first one presented on our home page. We analyzed what additional parameters were used to narrow query results, and found that while most of these were available on the homepage, some were not. To maximize the number of queries that can be performed in one stop, we added several features to the home page query (Figure 2). This included several ‘Finders’ that enable selection of terms utilizing a hierarchical tree structure driven by ontologies, search by synonyms, and autocomplete functionality. For example, where previously the IEDB homepage only allowed users to select ‘Class I’, ‘Class II’ or Nonclassical’ as the MHC restriction, now users can select any specific MHC allele, locus, haplotype or serotype for which the IEDB has data, based on the MHC Restriction Ontology (MRO) (3). In all, we now provide Finders for Organism, Antigen, Host, Assay, MHC and Disease on the redesigned IEDB homepage.

Next, we wanted to ensure that the values directly selectable by radio buttons on the home page are the most frequently queried ones. This led to a modification of the ‘host’ field to allow for direct selection of ‘mice’ over the previously available ‘rodents’. While we also noted that few queries were performed using the radio button for ‘non-peptidic epitopes’, we opted to maintain this direct selection to advertise that the IEDB contains non-peptidic epitope data, as this is a question we received in several user interactions. Overall, our optimized homepage search interface enables executing 98% of all past user queries directly from the home page, which exceeds our original goal of 95%.

Once a query is executed on the home page, results are displayed in a tabular format with all search parameters selected shown at the top of the results page as ‘Current Filters’. Note that by default a filter to select ‘Positive Assays Only’ is selected. Results can be further refined by filtering on additional parameters. For example, a user may start on the homepage with a general query, such as epitopes from a specified organism (e.g. Influenza A virus) and once the result page loads, they further narrow their results to epitopes restricted by a given MHC molecule, (e.g. ‘HLA-A*02:01’). Both query parameters, ‘Organism’ and ‘MHC Restriction’ are available on the homepage; however, typically users first perform a broad query and then further narrow it after viewing the results. We wanted to ensure that essentially all queries could be performed using the query results refinement mechanism and examined what parameters should be added that were only available in the ‘specialized search’ interfaces. Based on the query logs, we added the ability to search for epitopes with 3D structure data and search by post-translational modification of epitope residues, and added an entirely new search pane for antibody and TCR sequence data. These additions resulted in >99% of queries performed in the past through a variety of query mechanisms now being executable through the combined homepage search and result filtering.

We routinely assess how integrating data from external resources can be used to improve the search interface. One current example is our work on organizing protein sequences through the Protein Finder. Most of the >450 000 peptidic epitopes in the IEDB are described as being derived from specific proteins. Especially for viruses and bacteria, a large variety of protein isoforms exist in each species and it is important to note which variant is studied for immune recognition. At the same time, users want to retrieve results from different isoforms in one step. For example, there are more than 300 different hemagglutinin isoforms in which IEDB epitopes are described. To group different isoforms together for a given species, we align them to reference proteomes obtained from UniProt (4). Since the Protein finder was first implemented in 2015, there have been major additions to UniProt in terms of the number of reference proteomes available and how they are assembled. UniProt has also recently introduced the concept of ‘pan proteomes’ for species where there is a large degree of variability in the proteins encoded in different strains. This is a common issue in bacteria such as E. Coli, due to horizontal gene transfer where one strain may or may not contain a gene for antibiotic resistance. To take advantage of these new data in UniProt, we are implementing an automated process to update the choice of reference proteomes from UniProt to ensure that we utilize the best version for the IEDB dataset. This process also drives recuration of any data found to be in error. For example, we have observed that proteins for which only a single epitope has been curated in the IEDB are enriched for curation errors, which we are now reviewing for recuration. Thus, the ongoing protein tree revision also provides an opportunity to find and correct errors in the IEDB data.

## RESULTS PRESENTATION

We have traditionally provided query results in three main formats: (i) Results webpage tables displaying key values such as host and assay type, with summaries of more complex data, such as the immunization fields. (ii) Details webpages for assays that display most fields for which information is available and Details webpages for epitopes that provide information on the epitope and link to all assays. (iii) Spreadsheet exports of results containing many data field columns, populated or not. As a result of the newly added receptor sequence data, we added a results page tab to display a summary table of the receptor sequences relevant to the search parameters. Similarly, new receptor details pages and a new export table that contains this data were added.

**Figure 3.**
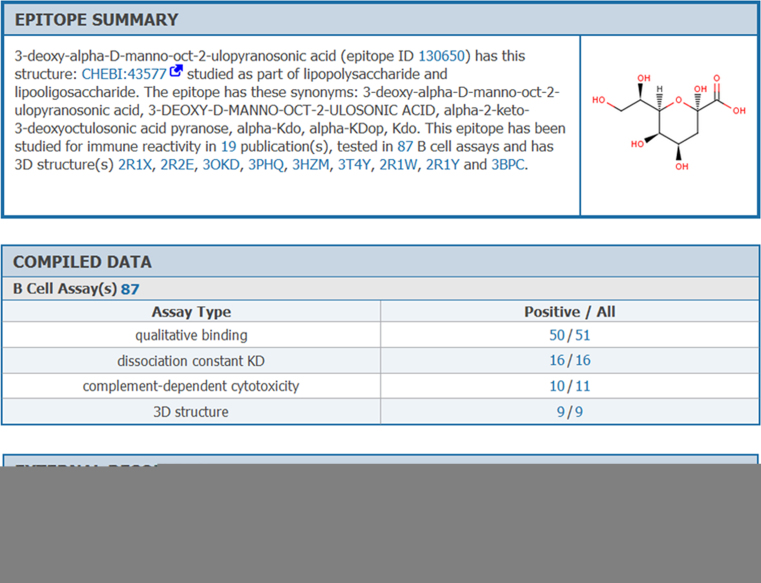
Redesigned Epitope Details page. The newly designed Epitope Details page includes information on all experimental contexts a structure was tested in and links out to all 3D structures demonstrating the binding of antibodies, TCRs or MHC molecules to the epitope.

As data has accumulated, it became apparent that we need to provide better aggregate summaries. Epitopes can be tested in hundreds or thousands of experiments. We wanted to provide an overview of the main findings that does not require users to browse through each individual experiment. Thus, we designed a new Epitope Details page, with a textual summary of the aggregated data. This summary includes information on all of the experimental contexts the structure was tested in and links out to all 3D structures demonstrating the binding of antibodies, TCRs or MHC molecules to the epitope, as shown in Figure 3. We also added new data tables to this webpage to present a summary of assay types each epitope was tested in, how often it was tested, the outcome, and links to these assays. As this data is compiled from the entirety of the literature, a user can now easily and quickly form opinions regarding each epitope structure, relevant to their specific research needs. For example, the assays performed on the epitope in Figure 3 suggest that the epitope causes complement dependent cellular cytotoxicity, as 10 of the 11 assays performed had positive outcomes.

Lastly, the revised epitope details page provides links to related resources, including the IEDB prediction tools and relevant external websites. We continually assess and update external links, looking for the development of new resources and ensuring existing links still resolve. We recently added links to the carbohydrate specific epitope resource Glycotoucan (5), in addition to the existing PubChem (6), IMGT (http://www.imgt.org), National Center for Biotechnology Information (NCBI) (7), UniProt (4) and Protein Data Bank (PDB) (8) links.

## USE OF ONTOLOGIES IN THE IEDB

The integration of formal ontologies into the IEDB has been ongoing for many years (9,10) to provide users with the accepted nomenclature for each data type, for example the organism names determined by NCBI Taxonomy (7) or the proper MHC terminology for each species provided by MRO. Ontologies also provide hierarchical structures to facilitate understanding and searching of data. For example, by using the Ontology of Biomedical Investigations (OBI) (11) to drive the IEDB Assay Finder, users can search for all T cell assays, all T cell assays measuring cytokines, or all T cell assays measuring a specific cytokine, such as IL-2 because OBI logically defines its assay terms accordingly. Ontological logical definitions also enable logical validation of data by flagging inconsistencies, as previously described (9,10).

The use of ontology terms has the added benefit of making IEDB data more interoperable with other projects using these same vocabularies. For ontologies already integrated into the IEDB, including OBI and Chemical Entities of Biological Interest (ChEBI) (12), we perform annual reviews to determine if our needs are being met, often resulting in the need for recuration, as well as new term requests being made to each ontology, resulting in improvements in both the IEDB and the originating ontology. Over the past 3 years, we have requested 20 new assays to be added to OBI and 1227 new structures added to ChEBI to describe data captured by the IEDB. We continue to expand our use of ontologies for more fields, and have recently incorporated the MRO, Uberon, cell type (13) and cell line ontologies (14). The ultimate goal is to have all terms utilized in the IEDB to be formally defined in ontologies.

## FAIR

A call for improved data guidelines for public data repositories in the form of the FAIR principals was recently established (15). We assessed how well the IEDB complied with the defined principals. If we found the IEDB to be lacking, we either made direct changes or implemented plans to reach those goals (16). This process resulted in improvements that benefit our users. For example, we now make formal identifiers for terms more accessible in the IEDB data exports and are in the process of adding this information to the assay details pages. We also added provenance and licensing information to the IEDB webpages, making our terms and conditions more transparent. Additionally, this assessment led us to make all our internal controlled vocabulary terms into publicly available ontology terms in our Ontology for Immune Epitopes (ONTIE, ontology.iedb.org). We built a web interface that provides additional information for these terms and we now publically share the IEDB specific vertebrate tree, that the IEDB has long used to extend NCBI taxonomy to accommodate laboratory animal strains often used in research, as previously described (17). Providing this information to the public enables interoperability and was partially driven by requests from external resource developers, but also serves our existing user community by making our practices more transparent. Going forward, we plan to continue to look for ways to become more FAIR compliant, including working with related resources such as the Human Immunology Project Consortium (HIPC) (https://www.immuneprofiling.org) and the National Institutes of Health (NIH) Bioinformatics Resource Centers (BRCs) (https://www.niaid.nih.gov/research/bioinformatics-resource-centers) to make related data fields semantically interoperable in a machine interpretable fashion.

## FUTURE PLANS

The IEDB plans to keep current with new literature as it is published, as well as to continue enhancing the website to meet user needs. Specifically, we are planning to assess feedback on the new receptor search interface, once users have had time to become familiar with its functionality. We will iteratively review ontology and external resource integration into the user interface and are currently working with ChEBI toward a revision of the hierarchy used by the IEDB search interface for non-peptidic epitopes, similar to the effort described for peptidic epitopes. With the integration of each new ontology into the IEDB, we gain greater interoperability with other resources. We plan to develop more complex ontology modelling of our data and have been testing these principles via an early stage triple store that presents IEDB data alongside that of related resources ImmPort (18) and PlasmoDB (19), allowing federated queries across the combined dataset. This integration also furthers our FAIR compliance. Additionally, we are working toward several other FAIR goals by standardizing how we describe the location within the journal article where data originated, working with public resources such as Wikidata (20) to better integrate IEDB content, and improving the RDF/OWL representation of the IEDB. We intend to anticipate needs of our user community and continually work toward improvements, with the ultimate goal of facilitating immunology research.
